# Depression symptoms and quality of life in patients receiving renal replacement therapy in Jordan: A cross-sectional study

**DOI:** 10.1016/j.amsu.2021.102384

**Published:** 2021-05-13

**Authors:** Suhaib Muflih, Karem H. Alzoubi, Sayer Al-Azzam, Belal Al-Husein

**Affiliations:** a Department of Clinical Pharmacy, Jordan University of Science and Technology, Irbid, Jordan; b Department of Pharmacy Practice and Pharmacotherapeutics, College of Pharmacy, University of Sharjah, Sharjah, United Arab Emirates

**Keywords:** Depression, Depressive symptoms, Psychological, Quality of life, ESRD, Renal replacement therapy, SF-36

## Abstract

Patients with chronic illnesses, such as those with chronic kidney disease (CKD) that are undergoing renal replacement therapy (RRT), face significant psychological changes. This descriptive cross-sectional research was carried out to investigate the factors that influence depressive symptoms and quality of life (QoL) in patients with end-stage renal disease. Data were collected from 70 participants undergoing RRT using a self-reported questionnaire that included sociodemographic information, depressive symptoms, disease status, and QoL. The Beck Depression Inventory-II (BDI-II) screening scale was used to measure depressive symptoms and the SF-36 (Medical Outcome Survey-Short Form 36) was used to assess QoL. Participants in the study rated their depressive symptoms as ‘minimum (44.3%), ‘mild’ (20%), ‘moderate’ (21.4%), and ‘severe (14.3%). Furthermore, a shorter duration of dialysis and comorbid conditions were significantly associated with the development of depressive symptoms. Patients on RRT for a longer period of time had lower physical activity scores than patients on dialysis for a shorter period of time. Male participants have a higher mental QoL than female participants, suggesting that the mental aspect of renal disease is less impaired than the physical aspect. The findings of this study are expected to increase awareness of RRT therapy targets and enhance patient outcomes.

## Highlights

• Patients who received dialysis for longer-duration reported less depression symptoms.
• There were no strong correlations between depression severity and age or gender.
• Patients who received RRT for a longer time had lower levels of physical activity.
• Moderate to severe depressive symptoms had an effect on mental health and vitality.

## 1 Introduction

Depression and anxiety are common psychiatric disorders among patients with end-stage renal disease (ESRD) [1,2]. Chronic kidney disease (CKD) affects more than 10% of adults worldwide, resulting in the deaths of nearly 5–10 million patients per year [3]. To better control the complications of CKD, the majority of patients in the final stage of the disease, ESRD, should undergo renal replacement therapy (RRT) such as renal dialysis or kidney transplantation [4]. Jordan's total prevalence rate of hemodialysis patients per million population (pmp) was 709 (4690 patients) in 2015, up from 406 (2666 patients) in 2008 and 312 pmp in 2002. (1659 patients). Jordan had a much higher prevalence of patients receiving RRT than Saudi Arabia (499 pmp), but it was lower than Lebanon (788 pmp), Finland (815 pmp), the United Kingdom (855 pmp), Spain (1090 pmp), France (1100 pmp), Canada (1200 pmp), and the United States (1200 pmp) (1850 pmp). There were 726 new cases of ESRD, with men having a higher rate of RRT than women (1.66:1, respectively) [5].

Since the possible advantage is often associated with side effects and risks, accepting RRT as a lifelong treatment could have negative psychological implications [2]. Furthermore, patients who have encountered negative RRT symptoms have often indicated a decrease in their quality of life (QoL) [6]. Previous research has shown that people with chronic diseases have a lower quality of life and more mental health problems, such as depression [7]. Calman described QoL as the degree to which our aspirations and desires are balanced with reality, and he proposed that the difference between patients' expectations and what actually happens in medical care be narrowed [8]. Depression is a mood disorder that has a detrimental effect on feelings of worthiness, sleepiness, appetite, sexual impulses, and other everyday activities [9]. According to the American Psychiatric Association, a number of studies have shown that CKD and ESRD patients suffer from depressive conditions such as persistent depressive disorder (PDD) and major depressive disorder (MDD). Patients with MDD and PDD can have common symptoms such as insomnia, hypersomnia, exhaustion, and a sense of worthlessness, but PDD patients have a long-term type of depression that lasts at least two years [9,10].

Furthermore, depression may have an effect on medical outcomes in ESRD patients by altering immunologic and stress responses, affecting nutritional status, and reducing adherence to dialysis and medical regimens [11]. Depression has also been associated with a lower quality of life and an increased risk of death in patients with CKD and ESRD [12,13]. Farrokhi et al. (2014) analyzed 31 studies and discovered that patients undergoing long-term RRT had a higher mortality risk if they had depressive symptoms [14].

In order to increase the importance and outcomes of RRT treatment, healthcare professionals need to know whether patients undergoing RRT are more likely to experience depressive symptoms and suffer from physical or mental limitations, and which variables may be correlated with known levels of depression and limited QoL. One of the major limitations of previous research is that the relationship between depressive symptoms and RRT has received little attention, especially in Jordan. The aim of this study is to look into the factors that are related to depressive symptoms and the QoL of patients in Jordan who are undergoing RRT, or central hemodialysis (HD). The authors hypothesize that overall health-related QoL, as assessed by the SF-36, would have a strong negative association with depressive symptoms scores among ESRD participants, so the findings of this study are expected to draw more attention to the psychological distress of patients undergoing RRT.

## 2 Materials and methods

### 2.1 Study design

A descriptive, cross-sectional study was conducted in Jordan to determine the factors that affect depressive symptoms and QoL in RRT patients, as well as the association between QoL and severity of these depressive symptoms and years of RRT, sociodemographic variables, and comorbidities. The current study was completed in accordance with the STROCSS guidelines [15]. The research was registered with the Research Registry and given a unique identifier (UIN): researchregistry6724, which can be found at https://www.researchregistry.com/browse-the-registry#home/.

### 2.2 Patient population and data collection procedures

From August to November 2018, the participants were recruited from the dialysis units at King Abdullah University Hospital (KAUH) in Irbid, Jordan. The current study's prospective participants were all 18 or older, diagnosed with ESRD, and had been on dialysis for at least 3 months prior to the start of the study. All qualified participants who decided to voluntarily participate in the study gave their informed written consent. Patients who had only undergone RRT once or who had received a kidney transplant were not included in the sample. Patients with a history of depression or who had undergone treatment for depression (n = 120) were also omitted from this study because their depression may be caused by a number of factors unrelated to renal disease. The principal investigator gathered a convenient sample and administered an anonymous self-reported questionnaire.

### 2.3 Instruments

The SF-36 (Medical Outcome Survey-Short Form 36) questionnaire was used to determine the QoL [16,17]. It consists of 36 elements that assess QoL in eight different dimensions: Physical Functioning (PF), Role Limitations Due to Physical Problems (RLPP), Role Limitations Due to Emotional Problems (RLEP), Vitality, Emotional Well-Being (EWB), Social Functioning (SF), Pain (PN), General Health (GH). The physical component of QoL is represented by PF, RLPP, RLEP, and vitality, while the mental component is represented by EWB, SF, PN, and GH. Items from the same scale should be averaged together to generate the subscale scores for each health concept answered by respondents, according to RAND's guidance on its version of the SF-36. The SF-36 items are rated on a scale of 0–100, with a higher score indicating better health [18]. The Beck Depression Inventory-II (BDI-II) scale was used to measure depressive symptoms, which is a self-reporting method for assessing depressive symptoms that is often used to determine mental health in ESRD patients. The total score ranges from 0 to 13 is considered minimal, with 14–19 being mild, 20 to 28 being moderate, and 29 to 63 being severe depressive symptoms [19].

### 2.4 Variables

⁃ The independent variables used to explain variations in QoL were sociodemographic variables (i.e., economic class, employment status, gender, age, marital status, comorbidities, and duration of treatment) and depressive symptoms.
⁃ The independent variables used to explain variations in depressive symptoms were sociodemographic variables and QoL.

The socioeconomic factor was classified and ranked according to the participants’ income into three groups (1 = best status (>1000 JOD), 2 = average (501–1000 JOD), and 3 = worse status (<500 JOD). The comorbidities reported by the participants were gathered into the Charlson Comorbidity Index (CCI), which is a continuous variable used to predict the one-year mortality among patients where a larger index number represents a larger number of comorbidities [20].

### 2.5 Statistical analysis

The basic features of the study were demonstrated using descriptive statistical analysis of the results, which included frequencies, means, standard deviations, and variances. Pearson correlation was used to assess the relationship between BDI-II scores and some health concepts related to QoL, as well as the relationship between BDI-II scores and years of dialysis. Prior to the analysis, the data were transformed to achieve normality using base 10 logarithms. The *t*-test and one-way analysis of variance (ANOVA) were used to measure patients' physical and mental scores and identify any significant differences across levels of independent variables using the statistical software IBM® SPSS® version 26.0. The F statistic values from the one-way ANOVA model were also used to evaluate patients' depressive symptom scores based on years of dialysis. Posthoc Tukey tests were used for pairwise comparisons (i.e., years of dialysis with depressive symptoms levels). The independent variables were then used in linear regression models to explain the variations in the dimensions of QoL. In all statistical analyses, *p*-values less than 0.05 were considered significant. In the regression equation, gender was viewed as a dummy variable with two attributes: male and female. The female was recoded as 1 as a dummy variable, while the male was recoded as 0. Table 1 shows the internal reliability (Cronbach's alpha) of all QoL subscales used in this study. Before the research began, the Institutional Review Board (IRB) at Jordan University of Science and Technology received and approved the study's proposal, an anonymous self-reported patient survey, and the informed consent form.

**Table 1:** SF-36 scores and internal consistency.

SF-36 subscales	No of items	Mean ± SD	Cronbach α
Physical Functioning	10	49.1 ± 31.7	0.91
Role Limitations Due to Physical Problems	4	30.1 ± 28.5	0.83
Role Limitations Due to Emotional Problems	3	43.5 ± 40.0	0.87
Vitality	4	47.4 ± 22.4	0.86
Emotional Well-Being	5	67.7 ± 21.1	0.73
Social Functioning	2	60.6 ± 28.5	0.78
Pain	2	59.9 ± 28.3	0.84
General Health	5	44.3 ± 19.7	0.81

## 3 Results

### 3.1 Study sample characteristics

A total of 70 patients with ESRD were included in the study (54% males and 46% females). At least one chronic condition was self-reported by 91%. The participants in this study were on dialysis three days a week. Chronic disease prevalence was increasing in older age groups, but it was fairly distributed between male and female groups. Table 2 shows the distribution of various demographic characteristics.

**Table 2:** Percentage distribution of selected demographic variables of patients in the sample (n = 70).

Variable	Frequency (%)
**Age**	
18-25	15 (21.4)
26-35	13 (18.6)
36-45	9 (12.9)
>45	33 (47.1)
**Gender**	
Male	38 (54.3)
Female	32 (45.7)
**Nationality**	
Jordanian	68 (97.1)
Others	2 (2.9)
**Marital Status**	
Single	46 (65.7)
Married	17 (24.3)
Divorced	7 (10.0)
**Educational Level**	
High School	12 (17.1)
Bachelor	20 (28.6)
Master	18 (25.7)
PhD	20 (28.6)
**Income**	
Low	38 (54.3)
Moderate	19 (27.1)
High	13 (18.6)
**Family Hx**	
Yes	5 (7.1)
No	61 (87.2)
Not Sure	4 (5.7)
**Smoker**	
Yes	60 (85.7)
No	10 (14.3)
**Obesity**	
Yes	19 (28)
No	51 (72)
**Hypertension**	
Yes	54 (77)
No	17 (23)
**Diabetes**	
Yes	27 (38.6)
No	43 (61.4)
**Cardiovascular Disease**	
Yes	29 (41.4)
No	41 (58.6)

### 3.2 Depressive symptoms in hemodialysis patients

The proportions of participants with minimum depressive symptoms (44.3%), mild (20%), moderate (21.4%), and severe depressive symptoms (14.3%) were recorded. BDI-II scores were found to have a substantially negative association with the following QoL health concepts using Pearson correlation: RLEP (p < 0.05), vitality (p 0.05), emotional well-being (p < 0.05), SF (p < 0.05), pain (p < 0.05), and general health (p < 0.05). However, there was no evidence of a connection between BDI-II scores and physical functioning or task limitations due to physical problems. Years of dialysis were also found to have a negative but significant association (p = 0.032) with total BDI-II scores, with more years of dialysis being associated with less depressive symptoms. Before conducting ANOVA tests, the data were transformed using base 10 logarithms to achieve normality. Patients who had been on dialysis for more than four years had substantially fewer depressive symptoms than those who had only been on RRT for up to two years, according to a posthoc Tukey test. Table 3 displays the F values that have been calculated. Patients who had been on dialysis for longer (4 years) were less likely to have other comorbidities, as measured by CCI, which may have affected the findings.

**Table 3:** Estimated F Statistic Values of the One-Way ANOVA Model Pertaining to Patients’ Depression Scores based on Years of Dialysis.

	Sum of Squares	df	Mean Square	F	Sig.
Between Groups	9.899	2	4.949	3.806*	.027
Within Groups	87.141	67	1.301		
Total	97.040	69			

*Significance at p < 0.05.

### 3.3 The health-related quality of life in hemodialysis patients

As shown in Table 4, Table 5, a one-way ANOVA was used to measure patients' physical and mental scores and identify any significant variations between levels of independent variables. Several components of physical QoL were found to vary significantly from the following variables: age, gender, marital status, obesity, dialysis period, and depressive symptoms variables. When compared to older patients, younger patients showed better physical functioning and role limitations due to physical problems. Male patients, as well as married patients, demonstrated a higher degree of vitality than their counterparts. Married patients have reported better role limitation scores as a result of physical problems than single patients. Patients who were younger, male, and married reported less pain than those who were older, female, and single or divorced. Obesity was associated with lower scores in role limitations due to physical problems scores. There were no major correlations between the severity of depressive symptoms and the patients' age groups or gender, on the other hand. Age, gender, marital status, education level, obesity, and depressive symptoms variables were also found to have significant differences with several components of mental QoL. When compared to older patients, younger patients reported higher social functioning scores. Patients with a higher level of education had considerably less pain than patients with a lower level of education. Obesity was related to lower levels of social functioning. The results also revealed that the dialysis period was significantly correlated with physical functioning, with longer dialysis duration resulting in worse physical functioning. Furthermore, patients with minimal to mild depressive symptoms scored significantly higher than patients with moderate to extreme depressive symptoms on the SF-36 subscales of vitality (p = 0.04), emotional well-being (p = 0.01), and general wellbeing (p = 0.03). As a result, patients with moderate to extreme depressive symptoms had worse mental wellbeing and vitality than those with minor symptoms.

**Table 4:** Relationships between physical dimensions of QoL and variables of interest.

Variable	PF		RLPP		RLEP		Vitality	
	Mean	SD	Mean	SD	Mean	SD	Mean	SD
**Age**								
18-25	0.630	0.235	0.709	0.203	0.632	0.175	0.489	0.212
26-35	0.562	0.211	0.488	0.172	0.523	0.166	0.663	0.242
36-45	0.523	0.195	0.372	0.116	0.422	0.225	0.564	0.327
>45	0.422	0.304	0.503	0.226	0.485	0.224	0.446	0.286
*p* value	0.03		0.01		0.09		0.15	
**Gender**								
Male	0.569	0.259	0.537	0.231	0.543	0.218	0.574	0.287
Female	0.487	0.304	0.524	0.220	0.489	0.203	0.437	0.248
*p* value	0.38		0.90		0.29		0.047	
**Marital Status**								
Single	0.487	0.259	0.478	0.189	0.487	0.219	0.546	0.288
Married	0.529	0.254	0.703	0.228	0.625	0.172	0.511	0.221
Divorced	0.490	0.411	0.466	0.246	0.463	0.179	0.270	0.205
*p* value	0.21		0.00		0.14		0.04	
**Educational Level**								
High School	0.473	0.371	0.538	0.233	0.466	0.218	0.395	0.304
Bachelor	0.453	0.222	0.540	0.237	0.446	0.207	0.498	0.320
Master	0.600	0.293	0.560	0.216	0.540	0.206	0.483	0.243
PhD	0.578	0.255	0.486	0.223	0.604	0.197	0.622	0.209
*p* value	0.36		0.80		0.32		0.18	
**Income**								
Low	0.559	0.266	0.532	0.226	0.535	0.214	0.481	0.261
Moderate	0.480	0.296	0.540	0.235	0.469	0.202	0.466	0.307
High	0.519	0.318	0.513	0.218	0.539	0.222	0.660	0.230
*p* value	0.76		0.93		0.54		0.12	
**Smoker**								
Yes	0.475	0.343	0.599	0.287	0.521	0.228	0.466	0.298
No	0.538	0.274	0.520	0.213	0.517	0.211	0.515	0.274
p value	0.24		0.33		0.76		0.57	
*p* value	0.71		0.62		0.53		0.16	
**Obesity**								
Yes	0.385	0.309	0.439	0.197	0.536	0.230	0.477	0.317
No	0.583	0.255	0.564	0.226	0.510	0.206	0.520	0.261
*p* value	0.19		0.04		0.56		0.66	
**Duration of Dialysis**								
1-2	0.691	0.258	0.569	0.235	0.546	0.215	0.517	0.258
3-4	0.494	0.352	0.457	0.225	0.455	0.185	0.556	0.353
>4	0.386	0.250	0.479	0.175	0.485	0.218	0.452	0.270
*p* value	0.041		0.10		0.42		0.64	
**Depressive symptoms**								
minimum	0.565	0.290	0.494	0.233	0.513	0.288	0.489	0.342
mild	0.549	0.232	0.498	0.210	0.502	0.241	0.562	0.213
Moderate	0.545	0.209	0.505	0.240	0.559	0.216	0.373	0.108
Severe	0.589	0.248	0.553	0.287	0.406	0.259	0.351	0.296
*p* value	0.82		0.71		0.07		0.04	

*Significance at *p* < 0.05; PF, Physical Functioning; RLPP: Role Limitations Due to Physical Problems; RLEP: Role Limitations Due to Emotional Problems.

**Table 5:** Relationships between mental dimensions of QoL and variables of interest.

Variable	EWB		SF		PN		GH	
	Mean	SD	Mean	SD	Mean	SD	Mean	SD
**Age**								
18-25	0.506	0.269	0.623	0.218	0.571	0.144	0.457	0.234
26-35	0.537	0.250	0.592	0.197	0.614	0.211	0.664	0.292
36-45	0.424	0.379	0.543	0.294	0.613	0.217	0.548	0.167
>45	0.518	0.274	0.406	0.269	0.406	0.312	0.456	0.289
*p* value	0.70		0.01		0.05		0.32	
**Gender**								
Male	0.552	0.270	0.495	0.265	0.612	0.241	0.535	0.282
Female	0.459	0.282	0.517	0.263	0.388	0.247	0.475	0.265
*p* value	0.18		0.80		0.01		0.32	
**Marital Status**								
Single	0.534	0.262	0.475	0.276	0.514	0.282	0.552	0.272
Married	0.456	0.268	0.633	0.205	0.600	0.185	0.446	0.239
Divorced	0.476	0.403	0.389	0.198	0.229	0.144	0.375	0.334
*p* value	0.31		0.06		0.01		0.64	
**Educational Level**								
High School	0.435	0.288	0.453	0.314	0.283	0.234	0.402	0.304
Bachelor	0.474	0.324	0.591	0.297	0.477	0.271	0.539	0.277
Master	0.541	0.282	0.451	0.259	0.611	0.234	0.551	0.282
PhD	0.558	0.217	0.504	0.177	0.577	0.239	0.499	0.246
*p* value	0.68		0.25		0.01		0.48	
**Income**								
Low	0.510	0.296	0.524	0.269	0.537	0.286	0.512	0.278
Moderate	0.485	0.284	0.442	0.255	0.450	0.249	0.477	0.306
High	0.539	0.220	0.546	0.257	0.502	0.242	0.538	0.219
*p* value	0.84		0.44		0.46		0.87	
**Smoker**								
Yes	0.528	0.381	0.438	0.343	0.431	0.254	0.454	0.292
No	0.505	0.260	0.517	0.248	0.519	0.270	0.516	0.272
*p* value	0.84		0.34		0.34		0.56	
**Obesity**								
Yes	0.495	0.291	0.385	0.260	0.445	0.229	0.462	0.289
No	0.513	0.276	0.549	0.252	0.528	0.279	0.523	0.259
*p* value	0.79		0.02		0.56		0.42	
**Duration of Dialysis**								
1-2	0.498	0.246	0.535	0.245	0.513	0.256	0.512	0.276
3-4	0.506	0.328	0.446	0.300	0.429	0.355	0.490	0.267
>4	0.538	0.338	0.464	0.285	0.546	0.233	0.536	0.291
*p* value	0.61		0.54		0.47		0.94	
**Depressive symptoms**								
minimum	0.629	0.257	0.590	0.241	0.584	0.251	0.613	0.195
mild	0.513	0.290	0.471	0.252	0.454	0.303	0.470	0.360
Moderate	0.404	0.221	0.408	0.248	0.492	0.228	0.414	0.217
Severe	0.295	0.235	0.442	0.313	0.362	0.278	0.376	0.342
*p* value	0.01		0.05		0.08		0.03	

*Significance at *p* < 0.05; EWB: Emotional Well-Being; SF: Social Functioning; PN: Pain, GH: General Health.

The strength of the relationship between the two dimensions of QoL and the variables of interest was predicted using a multiple linear regression model. Age, the severity of depressive symptoms, and the duration of dialysis were all found to be important predictors of the physical components of QoL. Furthermore, the relationship between CCI and depressive symptoms was investigated. Patients showed higher levels of depressive symptoms as their CCI score increased, but this correlation was not statistically significant. Furthermore, the mental portion of QoL was significantly affected by age, gender, CCI, and levels of depressive symptoms.

## 4 Discussion

This research examines the quality of life and the factors that influence depressive symptoms in patients with CKD/ESRD who are receiving RRT. Despite the fact that CKD affects up to 15% of adults in the United States [21], multiple study groups have estimated the prevalence of CKD in developing countries and discovered that 6.8% of Jordanians have CKD stages 3, 4, and 5 [22]. The prevalence of this debilitating disease is increasingly growing, with RRT being the mainstay of treatment, placing a greater strain on the healthcare system [23].

Patients receiving RRT had moderate to severe depressive symptoms in 21.4% and 14.3% of cases, respectively. The decreased physical and mental components of QoL may be explained by these states. In light of previous research, the current study indicates that patients who experienced moderate to severe depressive symptoms after beginning RRT could benefit from cognitive-behavioral therapy combined with physical activity services, which could potentially minimize anxiety and depression, and thereby enhance their physical and mental health outcomes [[24], [25], [26], [27]]. In this study, patients who had dialysis for a longer period of time had substantially less serious depressive symptoms than those who had just started receiving RRT. This may be explained by the fact that patients receiving RRT can experience depressive symptoms when they become less active in the group, but that as time passes, they may begin to adjust and adapt to their current clinical regimen, or that the decreased fluctuation in their biochemical parameters causes them to experience less depressive symptoms than when they first started receiving RRT. Other research, on the other hand, found that younger age, female gender, longer dialysis period, and comorbidities were the most important predictors of depression and depressive symptoms [28,29]. The lack of a correlation between depressive symptoms and age or gender in this study could suggest that RRT patients take each day as it comes.

Furthermore, the results of this study showed that as CCI scores increased, patients reported more but not significantly depressive symptoms, suggesting that certain chronic conditions have no direct involvement in depression. Surprisingly, patients who had received RRT for a longer period of time had lower physical capability. This functional decline may be caused by an inability to perform routine tasks that are necessary for sustaining self-dependence, as well as a fear of falling, which causes patients' physical function to deteriorate. The lack of correlation between years of RRT and other aspects of QoL may be attributed to patients' control over their condition and treatment, as well as their deep desire to accept the challenges of RRT.

Patients with moderate to severe depressive symptoms had worse QoL than patients with minimal to mild depressive symptoms, demonstrating the SF-36's strong discriminative abilities. This result was in line with previous studies, which found that deteriorating mental health was related to progressive functional impairment [30]. Male RRT patients scored higher in vitality and pain fortitude than female RRT patients in this study, which may be due to gender-related biological factors as well as different lifestyles and cultural norms. This finding is in line with the findings of a previous study that enrolled 85,052 people [31]. Furthermore, male participants had a stronger mental QoL than female participants, suggesting that the mental aspect of renal disease was less affected in males than the physical aspect. While remarkable gender equality has been achieved in Jordan, in which women and men have equal rights in terms of educational attainment, future jobs, and health [32], future studies are needed to establish the connection between gender equality and better well-being. Only physical functioning, role limitations due to physical problems and social functioning components of QoL were substantially influenced by age in favor of younger adults, reflecting their psychological capacity to cope with the RRT's health limitations in contrast to older patients. Patients with a higher level of education had considerably less pain than those with a lower level of education, which may indicate their ability to self-medicate effectively and achieve remission.

As previously suggested by Mucsi et al.(2008), the findings here presented evidence of a significant connection between obesity and poor quality of life and highlighted the significance of preventive measures to achieve full well-being and maintain high levels of QoL among patients with chronic diseases [33]. In line with previous studies, results here revealed an increase in mental QoL [34] and a decrease in physical QoL among patients receiving RRT over time [35]. However, the results of this study contradicted those of Barbosa et al. (2017), who were unable to find a clear correlation between QoL and the existence of comorbidities or dialysis length [36].

## 5 Limitations of the study

The convenience of the study, the limited sample size, and the exclusion of patients with depression (n = 120) may limit the findings' generalizability. Also, the findings may have been influenced by the underrepresentation of CKD in certain sociodemographic characteristics. Furthermore, since this is a cross-sectional analysis, no cause-and-effect relationship can be identified.

## 6 Future studies

While significant findings were published here that could help achieve the goals of RRT therapy and enhance patients' health outcomes, future studies should focus on recruiting a larger sample size of RRT patients as well as patients who undergo other forms of RRT, such as renal transplants, in order to obtain more representative results. It's also imperative to look at the benefits of antidepressants in patients with kidney disease, as well as to use a variety of depression scales.

## 7 Conclusion

This study highlighted the factors that influence depressive symptoms and QoL in Jordanian hemodialysis patients. The results revealed that RRT patients had both physical and psychological consequences. Patients on dialysis for a longer period of time, however, showed less severe depressive symptoms. Several sociodemographic variables were not found to be significant QOL predictors, contrary to previous research findings. A more integrative and comprehensive approach to care should be considered in order to aid patients in transitioning and adjusting to their treatment plan, as well as to increase their social support. Intervention programs with modules that recognize and manage anxiety and depression should be introduced to inform and improve the psychological well-being and QoL of RRT patients.

## Sources of funding

This work was supported by the Deanship of Scientific Research at 10.13039/501100004035Jordan University of Science and Technology.

The Deanship of Scientific Research at Jordan University of Science and Technology has no such involvement in the collection, analysis and interpretation of data, writing of the manuscript, and in the decision to submit the manuscript for publication.

## Ethical approval

Ethical approval was obtained from the Institutional Review Board (IRB) at Jordan University of Science and Technology.

## Consent

All qualified participants who decided to voluntarily participate in the study gave their informed written consent.

## Authors' contributions

Dr. Muflih served as the principal investigator (PI) and was responsible for the successful administrating and execution of the entire research project. The PI participated in creating the survey questionnaire, data collection, performing statistical analysis, summering the results, drafting, and final approval of the manuscript.

Al-Zoubi participated in study design, performing statistical analysis, interpretation of data, drafting, critically revising, and final approval of the manuscript.

Dr. Al-Azzam participated in data collection, drafting, critically revising, and final approval of the manuscript.

Dr. Al-Husein participated in data collection, the study design, interpretation of data, also in drafting, critically revising, and final approval of the manuscript.

## Trial registry number

1. Name of the registry: The Research Registry.
2. Unique Identifying number or registration ID: researchregistry6724.
3. Hyperlink to your specific registration (must be publicly accessible and will be checked): https://www.researchregistry.com/browse-the-registry#home/.

## Guarantor

Suhaib Muflih, PhD, PharmD.

## Data availability

The datasets generated during and/or analyzed during the current study are available from the corresponding author on reasonable request.

## Provenance and peer review

Not commissioned, externally peer-reviewed.

## Declaration of competing interest

The authors of this original work declare that they have all participated in the design, execution, reviewing, and analysis of the paper, and that they have approved the final version. Also, there are no conflicts of interest associated with this publication and no financial support that could have influences its reported results. This work is not under publication or consideration for publication elsewhere.

## References

[1] Kimmel P.L. (2001). Psychosocial factors in dialysis patients. Kidney Int..

[2] Cukor D. (2007). Psychosocial aspects of chronic disease: ESRD as a paradigmatic illness. J. Am. Soc. Nephrol..

[3] Luyckx V.A., Tonelli M., Stanifer J.W. (2018). The global burden of kidney disease and the sustainable development goals. Bull. World Health Organ..

[4] Abecassis M. (2008). Kidney transplantation as primary therapy for end-stage renal disease: a national kidney foundation/kidney disease outcomes quality initiative (NKF/KDOQI™) conference. Clin. J. Am. Soc. Nephrol..

[5] As-Sayaideh A.Q.S.A, Asad M. (2015). National Registry of End Stage Renal Disease. National Registry of End Stage Renal Disease. 2015. https://www.moh.gov.jo/Echobusv3.0/SystemAssets/25708778-d442-49b5-932d-f10c96601c2a.pdf.

[6] Valderrábano F., Jofre R., López-Gómez J.M. (2001). Quality of life in end-stage renal disease patients. Am. J. Kidney Dis..

[7] Theofilou P. (2011). Quality of life in patients undergoing hemodialysis or peritoneal dialysis treatment. J. Clin. Med. Res..

[8] Calman K.C. (1984). Quality of life in cancer patients--an hypothesis. J. Med. Ethics.

[9] Segal D.L. (2010). Diagnostic and statistical manual of mental disorders (DSM‐IV‐TR). The Corsini Encyclopedia of Psychology.

[10] Shirazian S. (2017). Depression in chronic kidney disease and end-stage renal disease: similarities and differences in diagnosis, epidemiology, and management. Kidney international reports.

[11] Rysz J. (2017). The effect of diet on the survival of patients with chronic kidney disease. Nutrients.

[12] Pratt L.A. (2016). Excess mortality due to depression and anxiety in the United States: results from a nationally representative survey. Gen. Hosp. Psychiatr..

[13] Lee Y.J. (2013). Association of depression and anxiety with reduced quality of life in patients with predialysis chronic kidney disease. Int. J. Clin. Pract..

[14] Farrokhi F. (2014). Association between depression and mortality in patients receiving long-term dialysis: a systematic review and meta-analysis. Am. J. Kidney Dis..

[15] Agha R., Abdall-Razak A., Crossley E., Dowlut N., Iosifidis C., Mathew G., for the STROCSS Group (2019). The STROCSS 2019 guideline: strengthening the reporting of cohort studies in surgery. Int. J. Surg..

[16] Ware J., Kosinski M., Keller S. (2001). SF-36 Physical and Mental Health Summary Scales. A User's Manual.

[17] RAND (2009). Medical Outcomes Study: 36-item Short Form Survey Scoring Instructions. http://www.rand.org/health/surveys_tools/mos/mos_core_36item_scoring.html.

[18] Care., R.H (2020). Scoring Rules for the RAND 36-Item Health Survey. https://www.rand.org/health-care/surveys_tools/mos/36-item-short-form/scoring.html.

[19] O'Donnell K., Chung J.Y.J.P., psychosomatics (1997). The Diagnosis of Major Depression in End-Stage Renal Disease.

[20] Charlson M.E. (1987). A new method of classifying prognostic comorbidity in longitudinal studies: development and validation. J. Chron. Dis..

[21] Control C.f.D., Prevention (2017). National Chronic Kidney Disease Fact Sheet, 2014.

[22] Khalil A.A. (2018). Under‐diagnosed chronic kidney disease in Jordanian adults: prevalence and correlates. J. Ren. Care.

[23] Tabriziani H., Lipkowitz M.S., Vuong N. (2017). Chronic kidney disease, kidney transplantation and oxidative stress: a new look to successful kidney transplantation. Clin. kidney J..

[24] Duarte P.S. (2009). Cognitive–behavioral group therapy is an effective treatment for major depression in hemodialysis patients. Kidney Int..

[25] Zhang M. (2014). Relation between anxiety, depression, and physical activity and performance in maintenance hemodialysis patients. J. Ren. Nutr..

[26] Lerma A. (2017). Brief cognitive behavioural intervention for depression and anxiety symptoms improves quality of life in chronic haemodialysis patients. Psychol. Psychother. Theor. Res. Pract..

[27] Hou Y. (2014). Effects of cognitive behavioral therapy on insomnia of maintenance hemodialysis patients. Cell Biochem. Biophys..

[28] Hedayati S.S. (2005). Physician-diagnosed depression as a correlate of hospitalizations in patients receiving long-term hemodialysis. Am. J. Kidney Dis..

[29] Lopes A.A. (2004). Screening for depression in hemodialysis patients: associations with diagnosis, treatment, and outcomes in the DOPPS. Kidney Int..

[30] Barry L.C. (2012). Association between indicators of disability burden and subsequent depression among older persons. J. Gerontol.: Biomed. Sci. Med. Sci..

[31] Tsang A. (2008). Common chronic pain conditions in developed and developing countries: gender and age differences and comorbidity with depression-anxiety disorders. J. Pain.

[32] USAID (2020). M. Promoting Gender Equality and Women's Empowerment. https://www.usaid.gov/jordan/gender-equality-womens-empowerment.

[33] Mucsi I. (2008). Co-morbidity and quality of life in chronic kidney disease patients. J. Nephrol..

[34] Mittal S.K. (2001). Self‐assessed physical and mental function of haemodialysis patients. Nephrol. Dial. Transplant..

[35] Shdaifat E.A., Manaf M.R.A. (2012). Quality of life among Jordanian patients on haemodialysis and their caregivers. BMC Public Health.

[36] Barbosa J.B.N. (2017). Quality of life and duration of hemodialysis in patients with chronic kidney disease (CKD): a cross-sectional study. Fisioterapia em Movimento.

